# Comprehensive Exploration of the Effects of miRNA SNPs on Monocyte Gene Expression

**DOI:** 10.1371/journal.pone.0045863

**Published:** 2012-09-21

**Authors:** Nicolas Greliche, Tanja Zeller, Philipp S. Wild, Maxime Rotival, Arne Schillert, Andreas Ziegler, Panos Deloukas, Jeanette Erdmann, Christian Hengstenberg, Willem H. Ouwehand, Nilesh J. Samani, Heribert Schunkert, Thomas Munzel, Karl J. Lackner, François Cambien, Alison H. Goodall, Laurence Tiret, Stefan Blankenberg, David-Alexandre Trégouët, Cardiogenics Consortium, Tony Attwood, Belz Stephanie, Peter Braund, Jessy Brocheton, Jason Cooper, Abi Crisp-Hihn, Patrick (formerly Linsel-Nitschke) Diemert, Nicola Foad, Tiphaine Godefroy, Jay Gracey, Emma Gray, Rhian Gwilliams, Susanne Heimerl, Jennifer Jolley, Unni Krishnan, Heather Lloyd-Jones, Ulrika Liljedahl, Ingrid Lugauer, Per Lundmark, Seraya Maouche, Jasbir S Moore, Montalescot Gilles, David Muir, Elizabeth Murray, Chris P Nelson, Jessica Neudert, David Niblett, Karen O’Leary, Helen Pollard, Carole Proust, Angela Rankin, Augusto Rendon, Catherine M Rice, Hendrik Sager, Jennifer Sambrook, Schmitz Gerd, Michael Scholz, Laura Schroeder, Jonathan Stephens, Ann-Christine Syvannen, Stefanie (formerlyGulde) Tennstedt, Chris Wallace

**Affiliations:** Department of Haematology, University of Cambridge, Long Road, Cambridge, CB2 2PT, UK and National Health Service Blood and Transplant, Cambridge Centre, Long Road, Cambridge, CB2 2PT, UK; Medizinische Klinik 2, Universität zu Lübeck, Lübeck Germany; Department of Cardiovascular Sciences, University of Leicester, Glenfield Hospital, Groby Road, Leicester, LE3 9QP, UK; INSERM UMRS 937, Pierre and Marie Curie University (UPMC, Paris 6) and Medical School, 91 Bd de l’Hôpital 75013, Paris, France; Juvenile Diabetes Research Foundation/Wellcome Trust Diabetes and Inflammation Laboratory, Department of Medical Genetics, Cambridge Institute for Medical Research, University of Cambridge, Wellcome Trust/MRC Building, Cambridge, CB2 0XY, UK; Department of Haematology, University of Cambridge, Long Road, Cambridge, CB2 2PT, UK and National Health Service Blood and Transplant, Cambridge Centre, Long Road, Cambridge, CB2 2PT, UK; Medizinische Klinik 2, Universität zu Lübeck, Lübeck Germany; Department of Haematology, University of Cambridge, Long Road, Cambridge, CB2 2PT, UK and National Health Service Blood and Transplant, Cambridge Centre, Long Road, Cambridge, CB2 2PT, UK; INSERM UMRS 937, Pierre and Marie Curie University (UPMC, Paris 6) and Medical School, 91 Bd de l’Hôpital 75013, Paris, France; Department of Cardiovascular Sciences, University of Leicester, Glenfield Hospital, Groby Road, Leicester, LE3 9QP, UK; The Wellcome Trust Sanger Institute, Wellcome Trust Genome Campus, Hinxton, Cambridge CB10 1SA, UK; The Wellcome Trust Sanger Institute, Wellcome Trust Genome Campus, Hinxton, Cambridge CB10 1SA, UK; Klinik und Poliklinik für Innere Medizin II, Universität Regensburg, Germany; Department of Haematology, University of Cambridge, Long Road, Cambridge, CB2 2PT, UK and National Health Service Blood and Transplant, Cambridge Centre, Long Road, Cambridge, CB2 2PT, UK; Department of Cardiovascular Sciences, University of Leicester, Glenfield Hospital, Groby Road, Leicester, LE3 9QP, UK; Department of Haematology, University of Cambridge, Long Road, Cambridge, CB2 2PT, UK and National Health Service Blood and Transplant, Cambridge Centre, Long Road, Cambridge, CB2 2PT, UK; Molecular Medicine, Department of Medical Sciences, Uppsala University, Uppsala, Sweden; Klinik und Poliklinik für Innere Medizin II, Universität Regensburg, Germany; Molecular Medicine, Department of Medical Sciences, Uppsala University, Uppsala, Sweden; Medizinische Klinik 2, Universität zu Lübeck, Lübeck Germany; INSERM UMRS 937, Pierre and Marie Curie University (UPMC, Paris 6) and Medical School, 91 Bd de l’Hôpital 75013, Paris, France; Department of Cardiovascular Sciences, University of Leicester, Glenfield Hospital, Groby Road, Leicester, LE3 9QP, UK; INSERM UMRS 937, Pierre and Marie Curie University (UPMC, Paris 6) and Medical School, 91 Bd de l’Hôpital 75013, Paris, France; Department of Haematology, University of Cambridge, Long Road, Cambridge, CB2 2PT, UK and National Health Service Blood and Transplant, Cambridge Centre, Long Road, Cambridge, CB2 2PT, UK; Department of Haematology, University of Cambridge, Long Road, Cambridge, CB2 2PT, UK and National Health Service Blood and Transplant, Cambridge Centre, Long Road, Cambridge, CB2 2PT, UK; Department of Cardiovascular Sciences, University of Leicester, Glenfield Hospital, Groby Road, Leicester, LE3 9QP, UK; Trium, Analysis Online GmbH, Hohenlindenerstr. 1, 81677, München, Germany; The Wellcome Trust Sanger Institute, Wellcome Trust Genome Campus, Hinxton, Cambridge CB10 1SA, UK; Department of Haematology, University of Cambridge, Long Road, Cambridge, CB2 2PT, UK and National Health Service Blood and Transplant, Cambridge Centre, Long Road, Cambridge, CB2 2PT, UK; Department of Cardiovascular Sciences, University of Leicester, Glenfield Hospital, Groby Road, Leicester, LE3 9QP, UK; INSERM UMRS 937, Pierre and Marie Curie University (UPMC, Paris 6) and Medical School, 91 Bd de l’Hôpital 75013, Paris, France; Department of Haematology, University of Cambridge, Long Road, Cambridge, CB2 2PT, UK and National Health Service Blood and Transplant, Cambridge Centre, Long Road, Cambridge, CB2 2PT, UK; European Bioinformatics Institute, Wellcome Trust Genome Campus, Hinxton, Cambridge, CB10 1SD, UK; The Wellcome Trust Sanger Institute, Wellcome Trust Genome Campus, Hinxton, Cambridge CB10 1SA, UK; Medizinische Klinik 2, Universität zu Lübeck, Lübeck Germany; Department of Haematology, University of Cambridge, Long Road, Cambridge, CB2 2PT, UK and National Health Service Blood and Transplant, Cambridge Centre, Long Road, Cambridge, CB2 2PT, UK; Institut für KlinischeChemie und Laboratoriums medizin, Universität, Regensburg, D-93053 Regensburg, Germany; Trium, Analysis Online GmbH, Hohenlindenerstr. 1, 81677, München, Germany; Medizinische Klinik 2, Universität zu Lübeck, Lübeck Germany; Department of Haematology, University of Cambridge, Long Road, Cambridge, CB2 2PT, UK and National Health Service Blood and Transplant, Cambridge Centre, Long Road, Cambridge, CB2 2PT, UK; Molecular Medicine, Department of Medical Sciences, Uppsala University, Uppsala, Sweden; Medizinische Klinik 2, Universität zu Lübeck, Lübeck Germany; Juvenile Diabetes Research Foundation/Wellcome Trust Diabetes and Inflammation Laboratory, Department of Medical Genetics, Cambridge Institute for Medical Research, University of Cambridge, Wellcome Trust/MRC Building, Cambridge, CB2 0XY, UK; 1 INSERM UMR_S 937, Pierre and Marie Curie University (UPMC, Paris 6), Paris, France; 2 Université Paris-Sud, Paris, France; 3 Department of General and Interventional Cardiology, University Heart Center Hamburg, Hamburg, Germany; 4 Departments of Medicine II, University Medical Center, Johannes Gutenberg University Mainz, Mainz, Germany; 5 Institut für Medizinische Biometrie und Statistik, Universität Lübeck, Lübeck, Germany; 6 Human Genetics, Wellcome Trust Sanger Institute, Hinxton, United Kingdom; 7 Universität zu Lübeck, Medizinische Klinik II, Lübeck, Germany; 8 Klinik und Poliklinik für Innere Medizin II, Universität Regensburg, Regensburg, Germany; 9 Department of Haematology, University of Cambridge and National Health Service Blood and Transplant, Cambridge, United Kingdom; 10 Department of Cardiovascular Sciences, University of Leicester, Leicester, United Kingdom; 11 National Institute for Health Research Biomedical Research Unit in Cardiovascular Disease, Glenfield Hospital, Leicester, United Kingdom; 12 Department of Clinical Chemistry, University Medical Center, Johannes Gutenberg University Mainz, Mainz, Germany; 13 ICAN Institute for Cardiometabolism And Nutrition, Pierre and Marie Curie University (UPMC, Paris 6), Paris, France; Children's Hospital Boston, United States of America

## Abstract

We aimed to assess whether pri-miRNA SNPs (miSNPs) could influence monocyte gene expression, either through marginal association or by interacting with polymorphisms located in 3'UTR regions (3utrSNPs). We then conducted a genome-wide search for marginal miSNPs effects and pairwise miSNPs × 3utrSNPs interactions in a sample of 1,467 individuals for which genome-wide monocyte expression and genotype data were available. Statistical associations that survived multiple testing correction were tested for replication in an independent sample of 758 individuals with both monocyte gene expression and genotype data. In both studies, the hsa-mir-1279 rs1463335 was found to modulate in *cis* the expression of *LYZ* and in *trans* the expression of *CNTN6*, *CTRC, COPZ2*, *KRT9*, *LRRFIP1*, *NOD1*, *PCDHA6*, *ST5* and *TRAF3IP2* genes, supporting the role of hsa-mir-1279 as a regulator of several genes in monocytes. In addition, we identified two robust miSNPs × 3utrSNPs interactions, one involving *HLA-DPB1* rs1042448 and hsa-mir-219-1 rs107822, the second the *H1F0* rs1894644 and hsa-mir-659 rs5750504, modulating the expression of the associated genes.

As some of the aforementioned genes have previously been reported to reside at disease-associated loci, our findings provide novel arguments supporting the hypothesis that the genetic variability of miRNAs could also contribute to the susceptibility to human diseases.

## Introduction

MicroRNAs (miRNAs) represent a class of small (∼19–29 nucleotides) non coding RNAs that participate in gene post-transcriptional regulation. By binding to complementary target sites that are mainly located in gene 3'UTR regions, miRNAs inhibit mRNA translation either via mRNA degradation or via repression of mRNA translation [1]. A complete or nearly complete match of the miRNA with its target sequence generally results in a decrease of gene expression while a mismatch lead to a repression of mRNA translation. In general, miRNAs participate in regulating the expression of genes located remote from their genomic sequence; however when miRNAs are located within gene introns they are highly likely to modulate the expression of the host gene [2], [3].

According to the latest miRNA reference database (miRBase release 18, www.mirbase.org) [4], it is estimated that more than 1,500 miRNAs could exist in humans. A given miRNA may have several mRNA targets and participates in the regulation of a network of genes with genomic sequence similarities [5]. Reciprocally, a given mRNA may harbour in its 3'UTR region several different miRNA target sites and then be under the control of a set of miRNAs. It is estimated that, overall, about 50% of the genome would be subject to regulation by miRNAs [6], [7], making them one of the most important component of a cell. It is then not surprising to find miRNAs associated with a large number of human diseases (∼300 diseases according to the human miRNA disease database [8]) including cardiovascular and metabolic disorders [9]–[12].

As with any genomic sequence, miRNAs are prone to nucleotide variations that may have non negligible effects. The presence of a single nucleotide polymorphism (SNP) in the long miRNA primary (pri-miRNA) may affect its maturation process, its expression or the binding of the mature form to its target, which would then influence the expression of the target genes [13], [14]. This is the case, for example, for rs11614913 located in the pri-miRNA-196. It is hypothesized that this SNP affects miR-196a-2 expression, alters the miRNA–target binding site and influences cancer risks [15], [16]. The existence of a SNP in the miRNA genomic sequence may create mature miRNA variants, named isomiRs, whose predicted targets could differ from the original miRNA's targets [17]. In addition, the expression of miRNAs is known to be regulated by transcriptional factors, and by polymorphisms within the transcription factor binding sites, which may then modulate miRNA expression [18]. Finally, the presence of a SNP in the miRNA target sequences could also influence the expression of the targeted mRNAs [19], [20]. As an example, the rs58186-C allele located in the 3'UTR region of the *AGTR1* gene has been shown to decrease the efficiency of the binding of miR-155 to this gene. leading to an increase in *AGTR1* expression [20].

In this study, we conducted a genome-wide investigation of the effect of pri-miRNA SNPs (miSNPs) on monocyte gene expression in a large epidemiological study of healthy subjects for whom genome-wide monocyte gene expressions and genotype data have been collected, as part of the Gutenberg Health Study [21]–[24]. We also conducted a genome-wide search for pair-wise interactions between miSNPs and SNPs located in 3'UTR regions (3utrSNPs). We reasoned that such investigation could help to identify novel miRNA-sensitive regulation of gene expression in a key cell type participating in several disease processes including inflammation, atherosclerosis and immunity [25]. miSNPs effects identified were further validated for replication in a second large monocyte expression dataset, the Cardiogenics Transcriptomic Study (CTS) [26].

## Results

The Gutenberg Health Study (GHS) comprised 1,467 individuals (750 men and 717 women) [23]. All these individuals were typed for common SNPs using the Affymetrix Genome-Wide Human SNP Array 6.0 and their monocyte expression profiles were obtained from the Illumina HT-12 v3 Beadchip. Detailed description of these genome-wide expression and genotype data has already been provided elsewhere [21]–[24].

### Probes and SNPs selection

The GRCH37 release of the Human reference genome and the 17^th^ release of the miRNA database [4] were used to identify SNPs located within pri-miRNA sequences and 3'UTR regions. The number of miSNPs genotyped in GHS, or that could be substituted according to the SNAP software [27] by a “proxy” genotyped SNP in strong correlation (when expressed in terms of a pairwise linkage disequilibrium (LD) r^2^greater than 0.90) was 294, representing 258 distinct miRNAs.

The pre-processing of the expression data (see Methods) identified 22,004 probes covering 15,786 genes of “perfect” quality score according to ReMOAT [28] and not harboring a SNP in their genomic sequence. These probes were then tested for association with all genotyped miSNPs.

The search for interactions between miSNPs and 3utrSNP was restricted to probes targeting genes known to contain SNPs in their 3'UTR region that were either directly genotyped in GHS, or tagged by genotyped SNPs (r^2^>0.90). This led to the selection of a subsample of 8,768 probes characterizing 6,147 genes. In these genes, the total number of 3utrSNPs (or “proxy”) that were further studied was 10,783. The distribution of the number of 3utrSNPs per gene is given in Table 1.

**Table 1:** Distribution of the number of 3utrSNPs (or proxy) in the 6,147 studied genes.

# 3utrSNPsper gene	1	2	3	4	5	6	7	8	9	10	11	12	13	14	18
# genes	3,435	1,438	670	313	138	80	35	17	7	4	5	1	1	2	1

Note that, in some instances, a genotyped SNP can serve as a proxy (r^2^ >0.90) for several 3utr SNPs. This explains why the total number of 3utr proxy SNPs that can be derived from this table (11,353  =  1×3,435 + 2×1,438 + 3*670 + .....) is slightly higher than the number of really studied SNPs (10,783).

### Association of miSNPs with gene expression

#### GHS discovery phase

This analysis can be viewed as an ancillary study of the whole genome-wide association study between all genotyped SNPs and all expressions already conducted in GHS and whose results can be found in a publicly available resource [23]. At the Bonferroni correction level of 7.73×10^−9^ (ie. 0.05/(294×22,004)), fifty-seven associations between miSNPs and gene expression were significant (Table S1). However, forty-eight of these associations implicated miSNPs proxies mapping the genomic region of the genes they were associated with. We interrogated the GHS express database to identify the SNPs showing the strongest association with the associated expression among those with p<5.50×10^−5^ and located within 1Mb of the probe genomic sequence, thereafter referred to as the best *cis* eSNPs [23]. In six cases, the miSNP proxies were the best *cis* eSNPs. After adjusting for the effect of the best *cis* eSNPs, most miSNPs association vanished and only seven (bold lines in Table S1) remained significant at p = 7.73×10^−9^. Most of these 48 *cis* miSNPs associations are then likely due to LD between miSNPs and “true” *cis* eSNPs. Nevertheless, this must be investigated in greater depth as in several examples the corresponding miRNA was located within an intron of the associated gene, and could therefore participate in the regulation of the host gene.

Of more interest are the nine genome-wide significant associations that involved a miSNP located on a chromosome distinct from the one mapped by the associated gene, so called *trans* associations referring to associations involving SNPs that are located more than 1Mb away, or a distinct chromosome, from the associated probe. As shown in Table 2, the hsa-mir-1279 SNP rs1463335, tagged by the SNP rs317657 (r^2^ = 1.0), was associated in *cis* with expression of *LYZ* (R^2^ = 20.1%; p = 1.36×10^−76^) and *YEATS4* (R^2^ = 13.1%; p = 1.32×10^−46^), and in *trans* with expression of *CNTN6* (R^2^ = 3.3%; p = 1.16×10^−12^), *CTRC* (R^2^ = 3.5%; p = 1.39×10^−13^), *COPZ2* (R^2^ = 3.0%; p = 2.33×10^−11^), *KRT9* (R^2^ = 4.5%; p = 1.15×10^−15^), *LRRFIP1* (R^2^ = 10.0%; p = 1.50×10^−35^), *NOD1* (R^2^ = 2.1%; p = 7.25×10^−9^), *PCDHA6* (R^2^ = 9.2%; p = 9.44×10^−33^), *ST5* (R^2^ = 5.1%; p = 2.05×10^−18^) and *TRAF3IP2* (R^2^ = 4.9%; p = 2.74×10^−17^). It is of note that whereas the rs317657-C allele, with minor allele frequency 0.46, was associated with increased expression of *LYZ*, *YEATS4* and *NOD1*, it was associated with decreased levels of *CNTN6, CTRC, COPZ2, KRT9, LRRFIP1, PCDHA6, ST5* and *TRAF3IP2* expression. After adjusting for the best *LYZ cis* eSNP, the association of rs317657 with *LYZ* expression still retained genome-wide significance (p = 6.17×10^−11^) while the association with *YEATS4* disappeared (p = 0.734) (Table S1). According to the TargetScan bioinformatics tool [5], the position 648 to 654 of the 3'UTR *LYZ* region is predicted to be complementary at 8 bases with the hsa-mir-1279 sequence. This type of matching configuration, called 8mer, is usually considered to be a good prior for predicting potential targets of miRNA. After adjusting for *LYZ* expression, the *trans* association observed with rs317657 were reduced, but remained highly significant, p = 3.88×10^−11^, p = 1.15×10^−7^, p = 2.52×10^−6^, p = 1.65×10^−10^, p = 7.16×10^−29^, p = 2.44×10^−5^, p = 8.23×10^−28^, p = 1.81×10^−13^, p = 5.66×10^−10^ for *CNTN6*, *CTRC*, *COPZ2*, *KRT9*, *LRRFIP1*, *NOD1*, *PCDHA6*, *ST5* and *TRAF3IP2*, respectively. Corresponding p-values for the *trans* associations adjusted for *YEATS4* expression were p = 1.86×10^−9^, p = 1.72×10^−11^, p = 6.45×10^−9^, p = 9.48×10^−12^, p = 6.10×10^−28^, p = 3.76×10^−13^, p = 1.59×10^−28^, p = 2.33×10^−13^, p = 5.10×10^−8^, respectively. When the *trans* associations were adjusted for both *LYZ* and *YEATS4* expressions, they were hardly modified, with p-values ranging between p = 2.98×10^−6^ (*COPZ2*) to p = 6.55×10^−27^ (*PCDHA6*). As indicated in Table 3, these nine genes were not strongly correlated with each other, nor with expression of LYZ, the gene in which the rs31757 SNP was located.

**Table 2:** *Cis* and *trans*-associations observed with the hsa-mir-1279 rs1463335(1).

Associated Gene Expression					GHS			CTS		
Probe	Gene	CHR	Start	End	β(2)	SE	P(3)	β(2)	SE	P(3)
ILMN_1748730	CTRC	1	15764937	15773152	−0.03	0.004	1.39 10^−13^	−0.06	0.007	1.54 10^−15^
ILMN_2252021	LRRFIP1	2	238536223	238690289	−0.05	0.004	1.50 10^−35^	−0.12	0.010	6.65 10^−32^
ILMN_1699317	CNTN6	3	1134628	1445277	−0.02	0.003	1.16 10^−12^	−0.04	0.006	7.56 10^−12^
ILMN_1740494	PCDHA6	5	140207649	140391928	−0.04	0.003	9.44 10^−33^	−0.10	0.008	2.67 10^−31^
ILMN_1663381	TRAF3IP2	6	111880142	111927320	−0.03	0.003	2.74 10^−17^	−0.06	0.007	5.23 10^−17^
ILMN_2114422	NOD1	7	30464142	30518392	0.05	0.008	7.25 10^−9^	0.12	0.013	7.83 10^−19^
ILMN_1731063	ST5	11	8714898	8932497	−0.06	0.007	2.05 10^−18^	−0.22	0.019	2.51 10^−30^
ILMN_1815205	LYZ(1)	12	69742133	69748012	0.20	0.010	1.36 10^−76^	NA	NA	NA
ILMN_1801387	YEATS4(1)	12	69753531	69784575	0.15	0.010	1.32 10^−46^	0.19	0.020	3.27 10^−21^
ILMN_1792568	KRT9	17	39722092	39728309	−0.04	0.006	1.15 10^−15^	−0.11	0.016	1.11 10^−11^
ILMN_1667361	COPZ2	17	46103532	46115151	−0.03	0.005	2.33 10^−11^	−0.10	0.011	2.06 10^−18^

(1) The rs1463335 was tagged by the rs317657 and rs998022 in GHS and CTS, respectively. The rs146335 is located on chromosome 12, at position 69,667,075. As a consequence, the association observed with LYZ and YEATS4 are considered as *cis*-associations, the remaining eight as trans-associations.

(2) Regression coefficient associated with the rare miSNP allele under an additive effect model, adjusted for age and gender

(3) P-value of the association between miSNP and gene expression

**Table 3:** Correlation between gene expressions influenced by the rs317657 tagging the hsa-mir-1279 rs1463335.

	CTRC	LRRFIP1	CNTN6	PCDHA6	TRAF3IP2	NOD1	ST5	LYZ	YEATS4	KRT9
LRRFIP1	0.204	1								
CNTN6	0.137	0.237	1							
PCDHA6	0.202	0.449	0.200	1						
TRAF3IP2	0.129	0.271	0.202	0.270	1					
NOD1	0.225	−0.126	0.047	−0.062	0.029	1				
ST5	0.210	0.517	0.192	0.411	0.274	−0.176	1			
LYZ	−0.156	−0.143	−0.070	−0.125	−0.170	0.113	−0.125	1		
YEATS4	−0.079	−0.162	−0.110	−0.113	−0.250	−0.070	−0.140	0.558	1	
KRT9	0.217	0.485	0.168	0.402	0.302	−0.166	0.740	−0.133	−0.121	1
COPZ2	0.188	0.400	0.131	0.341	0.236	−0.140	0.592	−0.143	−0.093	0.590

#### Replication in CTS

We focused on the genome-wide significant *trans* associations observed with the hsa-mir-1279 miSNP proxy. These associations were tested for replication in CTS where monocyte expression was measured in a sample of 395 healthy individuals and 363 patients with coronary artery disease [26]. In CTS, the hsa-mir-1279 rs1463335 proxy was the rs998022 (r^2^ = 0.90). Its pairwise r^2^ with the GHS rs317657 proxy was 0.84. The probe tagging the *LYZ* gene expression was not available in CTS, but all other associations were replicable. As indicated in Table 2, they all replicated with consistent pattern of association as in GHS. The rs998022-G allele tagging the rs317657-C allele was associated with increased expression of *YEATS4* (R^2^ = 11.2%; p = 3.21×10^−21^) and *NOD1* (R^2^ = 9.82%; p = 7.83×10^−19^), but with decreased expression of *CNTN6* (R^2^ = 5.9%; p = 7.56×10^−12^), *CTRC* (R^2^ = 8.1%; p = 1.54×10^−15^), *COPZ2* (R^2^ = 9.7%; p = 2.06×10^−18^), *KRT9* (R^2^ = 5.9%; p = 1.11×10^−11^), *LRRFIP1* (R^2^ = 16.7%; p = 6.65×10^−32^), *PCDHA6* (R^2^ = 16.4%; p = 2.67×10^−31^), *ST5* (R^2^ = 17.0%; p = 2.51×10^−30^) and *TRAF3IP2* (R^2^ = 8.9%; p = 5.23×10^−17^). Associations were homogeneously observed in CAD patients and healthy subjects from CTS (Table S2).

### Search for miSNP × 3utrSNP interactions

#### GHS discovery phase

Each 3utrSNP was tested for interaction with all miSNPs with respect to the expression levels of the probes tagging the 3utrSNP-associated gene. Interactions were assessed using a standard linear regression analysis where both SNPs coded as 0,1,2 were included to the model together with the corresponding interaction term. Analyses were adjusted for age and sex. The total number of tested interactions was 4,890,102.

Instead of applying the standard Bonferroni correction to handle multiple testing, we followed the suggestion by Pare et al. [29] and adopted a weighted-Bonferroni correction according to the p-value of the Levene's test. This consists in prioritizing 3utrSNPs according to the significance of the test for a difference in the variance of expressions according to genotypes. This strategy relies on the statistical property that a significant difference in phenotypic variances according to sub-groups could be a marker for interaction phenomena.

Using this weighted-Bonferroni correction, 51 miSNP × 3utrSNP interactions were genome-wide significant at p<1.02×10^−8^ (Table 4). Note, only 31 would have been declared significant according the standard Bonferroni procedure (Table 4). Seventeen of the detected interactions involved the *RFPL1* rs13053624 that was found to interact with 17 miSNPs over 16 distinct miRNAs to modulate *RFPL1* expression (probe ILMN_1797383). One of these interacting miRNAs was hsa-mir-3674. Interestingly, according to microSNiPer database [30], *RFPL1* is predicted to harbor a SNP, rs13053817, in a potential target site for hsa-mir-3674 that is, according to the SNAP database, in nearly complete association with the identified rs13053624 (r^2^ = 0.90). No other strong biological and bioinformatics evidence could be obtained from public databases (miRanda [31], TargetScan [5], DianaMicro [32], PicTar [33], mirBase [4]) in favour of the 30 other genes we identified through our interaction search (Table 4).

**Table 4:** Genome-wide significant (p<1.02 10^−8^) interactions between miSNPs and 3utrSNPs on monocyte gene expression in the Gutenberg Health Study.

							GHS				
Gene	CHR	Probe	3utrSNP	miRNA	CHR	miSNP	miProxy	3utrProxy	P^(1)^	LeveneP-value	weighted P^(2)^
RFPL1	22	ILMN_1797383	rs13053624	hsa-mir-592	7	rs11563750	rs11563505	rs13053817	1.04 10^−35^	3.22 10^−5^	1.50 10^−36^
RFPL1	22	ILMN_1797383	rs13053624	hsa-mir-3920	11	rs12275715	rs12283329	rs13053817	1.21 10^−26^	3.22 10^−5^	1.74 10^−27^
TXNDC5	6	ILMN_1769082	rs8643	hsa-mir-125b-2	21	rs2823897	rs2211981	rs8643	8.95 10^−18^	3.39 10^−1^	1.23 10^−17^
TXNDC5	6	ILMN_1769082	rs1043784	hsa-mir-125b-2	21	rs2823897	rs2211981	rs3734589	1.26 10^−17^	3.18 10^−1^	1.64 10^−17^
LYZ	12	ILMN_1815205	rs710794	hsa-mir-1279	12	rs1463335	rs317657	rs710794	4.13 10^−15^	4.51 10^−23^	1.20 10^−16^
ASB1	2	ILMN_1683096	rs1044561	hsa-mir-125b-2	21	rs2823897	rs2211981	rs2334004	1.45 10^−16^	8.91 10^−1^	1.87 10^−15^
RFPL1	22	ILMN_1797383	rs13053624	hsa-mir-4656	7	rs3750013	rs17135110	rs13053817	2.28 10^−14^	3.22 10^−5^	3.29 10^−15^
ASB1	2	ILMN_1683096	rs2278768	hsa-mir-3119-1	1	rs17349873	rs1330387	rs2278768	3.71 10^−14^	1.34 10^−6^	4.10 10^−15^
RFPL1	22	ILMN_1797383	rs13053624	hsa-mir-30c-1	1	rs16827546	rs16827546	rs13053817	2.89 10^−14^	3.22 10^−5^	4.16 10^−15^
ECE1	1	ILMN_1672174	rs3026907	hsa-mir-1307	10	rs7911488	rs2271751	rs9287035	2.98 10^−13^	9.07 10^−46^	4.29 10^−15^
RFPL1	22	ILMN_1797383	rs13053624	hsa-mir-125b-1	11	rs2081443	rs2081443	rs13053817	2.40 10^−13^	3.22 10^−5^	3.47 10^−14^
PKD1L2	16	ILMN_1742788	rs1901818	hsa-mir-4272	3	rs9868022	rs9868022	rs7198127	8.92 10^−14^	8.80 10^−2^	5.47 10^−14^
ECE1	1	ILMN_1672174	rs3026907	hsa-mir-4670	9	rs2104533	rs2296666	rs9287035	5.16 10^−12^	9.07 10^−46^	7.42 10^−14^
ASB1	2	ILMN_1683096	rs2278768	hsa-mir-125b-2	21	rs2823897	rs2211981	rs2278768	5.30 10^−12^	1.34 10^−6^	5.85 10^−12^
RFPL1	22	ILMN_1797383	rs13053624	hsa-mir-4300	11	rs11603185	rs7944477	rs13053817	2.02 10^−11^	3.22 10^−5^	2.92 10^−12^
SPRY1	4	ILMN_2329914	rs300574	hsa-mir-4666	1	rs16841344	rs4653963	rs300555	1.52 10^−11^	1.16 10^−2^	5.10 10^−12^
HLA-DPB1	6	ILMN_1749070	rs1042448	hsa-mir-219-1	6	rs107822	rs213208	rs3128923	1.26 10^−10^	4.11 10^−8^	1.11 10^−11^
ASB1	2	ILMN_1683096	rs2278768	hsa-mir-4636	5	rs257095	rs6555591	rs2278768	1.09 10^−10^	1.34 10^−6^	1.20 10^−11^
RFPL1	22	ILMN_1797383	rs13053624	hsa-mir-4292	9	rs2811749	rs2811749	rs13053817	1.98 10^−10^	3.22 10^−5^	2.86 10^−11^
RFPL1	22	ILMN_1797383	rs13053624	hsa-mir-624	14	rs11156654	rs11156654	rs13053817	2.20 10^−10^	3.22 10^−5^	3.18 10^−11^
GPRC5C	17	ILMN_1724211	rs2706527	hsa-mir-3667	22	rs135771	rs135775	rs2706526	5.46 10^−9^	5.08 10^−79^	4.52 10^−11^
H1F0	22	ILMN_1757467	rs1894644	hsa-mir-659	22	rs5750504	rs2899293	rs763137	2.98 10^−10^	1.30 10^−1^	2.18 10^−10^
ECE1	1	ILMN_1672174	rs3026907	hsa-mir-548n	7	rs1649215	rs1637670	rs9287035	1.64 10^−8^	9.07 10^−46^	2.37 10^−10^
RFPL1	22	ILMN_1797383	rs13053624	hsa-mir-521-1	19	rs4803178	rs4803178	rs13053817	2.88 10^−9^	3.22 10^−5^	4.16 10^−10^
GPRC5C	17	ILMN_2352090	rs2706527	hsa-mir-3667	22	rs135771	rs135775	rs2706526	1.06 10^−7^	6.63 10^−102^	6.80 10^−10^
GPRC5C	17	ILMN_2352090	rs2706527	hsa-mir-107	10	rs17481096	rs17481096	rs2706526	1.20 10^−7^	6.63 10^−102^	7.69 10^−10^
HLA-DPB1	6	ILMN_1749070	rs1042448	hsa-mir-219-1	6	rs213210	rs213210	rs3128923	8.98 10^−9^	4.11 10^−8^	7.88 10^−10^
MXRA7	17	ILMN_1743836	rs10473	hsa-mir-490	7	rs6963819	rs2350780	rs7221855	2.66 10^−7^	6.10 10^−167^	1.04 10^−9^
SPRY1	4	ILMN_1651610	rs300574	hsa-mir-4666	1	rs16841344	rs4653963	rs300555	3.82 10^−9^	6.28 10^−3^	1.12 10^−9^
RFPL1	22	ILMN_1797383	rs13053624	hsa-mir-1236	6	rs403569	rs550513	rs13053817	7.89 10^−9^	3.22 10^−5^	1.14 10^−9^
GPRC5C	17	ILMN_2352090	rs2706527	hsa-mir-941-1	20	rs2427555	rs2427554	rs2706526	2.03 10^−7^	6.63 10^−102^	1.30 10^−9^
POGZ	1	ILMN_2329309	rs3811409	hsa-mir-4666	1	rs16841344	rs4653963	rs3811409	2.24 10^−9^	1.12 10^−1^	1.53 10^−9^
RFPL1	22	ILMN_1797383	rs13053624	hsa-mir-4643	6	rs16884450	rs16884450	rs13053817	1.28 10^−8^	3.22 10^−5^	1.85 10^−9^
ASB1	2	ILMN_1683096	rs1044561	hsa-mir-3973	11	rs262404	rs16928224	rs2334004	1.60 10^−10^	8.91 10^−1^	2.06 10^−9^
RFPL1	22	ILMN_1797383	rs13053624	hsa-mir-3646	20	rs11574730	rs11574730	rs13053817	1.70 10^−8^	3.22 10^−5^	2.45 10^−9^
ECE1	1	ILMN_1672174	rs3026907	hsa-mir-4460	5	rs13171514	rs13171514	rs9287035	2.47 10^−7^	9.07 10^−46^	3.55 10^−9^
RFPL1	22	ILMN_1797383	rs13053624	hsa-mir-3674	8	rs7003112	rs6558541	rs13053817	2.55 10^−8^	3.22 10^−5^	3.67 10^−9^
RFPL1	22	ILMN_1797383	rs13053624	hsa-mir-1205	8	rs9649959	rs9649959	rs13053817	2.78 10^−8^	3.22 10^−5^	4.02 10^−9^
RFPL1	22	ILMN_1797383	rs13053624	hsa-mir-4656	7	rs17829969	rs17829969	rs13053817	2.82 10^−8^	3.22 10^−5^	4.07 10^−9^
ECE1	1	ILMN_1672174	rs3026907	hsa-mir-4784	2	rs6709245	rs12463867	rs9287035	3.22 10^−7^	9.07 10^−46^	4.63 10^−9^
AAK1	2	ILMN_1880387	rs13427243	hsa-mir-3667	22	rs135771	rs135775	rs13427243	7.28 10^−9^	1.04 10^−1^	4.80 10^−9^
RFPL1	22	ILMN_1797383	rs13053624	hsa-mir-604	10	rs2368392	rs3758371	rs13053817	3.69 10^−8^	3.22 10^−5^	5.32 10^−9^
ECE1	1	ILMN_1672174	rs3026907	hsa-mir-215	1	rs3820455	rs34406824	rs9287035	3.88 10^−7^	9.07 10^−46^	5.58 10^−9^
RBM12	20	ILMN_1670841	rs6060539	hsa-mir-4755	20	rs2284385	rs2284390	rs2425125	4.06 10^−7^	1.65 10^−47^	5.62 10^−9^
ECE1	1	ILMN_1672174	rs3026907	hsa-mir-2113	6	rs9375085	rs9375085	rs9287035	4.02 10^−7^	9.07 10^−46^	5.79 10^−9^
RFPL1	22	ILMN_1797383	rs13053624	hsa-mir-1269b	17	rs7210937	rs2240567	rs13053817	4.93 10^−8^	3.22 10^−5^	7.10 10^−9^
ECE1	1	ILMN_1672174	rs3026907	hsa-mir-4705	13	rs7337292	rs7337292	rs9287035	5.10 10^−7^	9.07 10^−46^	7.33 10^−9^
PKD1L2	16	ILMN_1742788	rs1901818	hsa-mir-4473	9	rs16938058	rs16938057	rs7198127	1.24 10^−8^	8.80 10^−2^	7.60 10^−9^
MRPL43	10	ILMN_1678974	rs2295716	hsa-mir-608	10	rs4919510	rs4919510	rs3824783	3.06 10^−7^	9.68 10^−22^	9.44 10^−9^
ECE1	1	ILMN_1672174	rs3026907	hsa-mir-520d	19	rs2217653	rs9304754	rs9287035	6.62 10^−7^	9.07 10^−46^	9.52 10^−9^
ASB1	2	ILMN_1683096	rs1044561	hsa-mir-4636	5	rs257095	rs6555591	rs2334004	7.57 10^−10^	8.91 10^−1^	9.74 10^−9^

(1) P-value of the interaction test derived from the standard linear regression analysis

(2) P-value of the interaction test obtained when the Levene test p-value was used under a weighted-Bonferroni framework.

#### Replication in CTS

The fifty-one genome-wide significant interactions were tested for replication in CTS. However, only eight interactions could be replicable, which did not include the aforementioned interaction involving *RFPL1* rs13053624.

Using the same linear regression model, further adjusted for disease status as for the discovery phase, two interactions replicated in CTS at the Bonferroni-corrected level of 6.25×10^−3^ (Table 5).

**Table 5:** Replication in Cardiogenics of the miSNPs × 3utrSNPs detected in Gutenberg Health Study.

MiSNP×3utrSNP	rs17349873rs2278768	rs107822rs1042448	rs257095rs2278768	rs5750504rs1894644	rs6963819rs10473	rs262404rs1044561	rs2284385rs6060539	rs257095rs1044561
miRNA(CHR)	hsa-mir-3119-1(1)	hsa-mir-219-1(6)	hsa-mir-4636(5)	hsa-mir-659(22)	hsa-mir-490(7)	hsa-mir-3973(11)	hsa-mir-4755(20)	hsa-mir-4636(5)
Gene(CHR)	ASB1(2)	HLA-DPB1(6)	ASB1(2)	H1F0(22)	MXRA7(7)	ASB1(2)	RBM12(20)	ASB1(2)
Probe	ILMN_1683096	ILMN_1749070	ILMN_1683096	ILMN_1757467	ILMN_1743836	ILMN_1683096	ILMN_1670841	ILMN_1683096
Gutenberg Health Study								
Proxies	rs1330387rs2278768	rs213208rs3128923	rs6555591rs2278768	rs2899293rs763137	rs2350780rs7221855	rs16928224rs2334004	rs2284390rs2425125	rs6555591rs2334004
β^ (1)^	−0.480	−0.165	−0.233	−0.194	−0.065	0.988	0.164	0.375
WeightedP-value ^(2)^	4.10 10^−15^	1.11 10^−11^	1.20 10^−11^	2.18 10^−10^	1.04 10^−9^	2.06 10^−9^	5.62 10^−9^	9.74 10^−9^
Cardiogenics Transcriptomic Study								
Proxies	rs6703198rs10084192	rs439205rs3117222	rs257095rs10084192	rs6000905rs1894644	rs2350780rs9910052	rs262407rs10084192	rs2038123rs6121015	rs257095rs10084192
β^ (1)^	0.093	−0.274	0.045	−0.268	0.011	−0.025	0.099	0.045
P-value ^(3)^	4.62 10^−1^	**2.03 10^−13^**	5.18 10^−1^	**1.37 10^−8^**	5.98 10^−1^	8.29 10^−1^	7.22 10^−2^	5.18 10^−1^

(1) Regression coefficient of the interaction term when both miSNP and 3utr proxy SNPs coded 0/1/2 according to the number of carried rare alleles are introduced in a linear regression model together with their interaction term.

(2) P-value of the interaction test obtained in GHS when the Levene test p-value was used under a weighted-Bonferroni framework.

(3) P-value of the interaction test derived from the standard linear regression analysis in Cardiogenics. Bold p-values are significant after Bonferroni correction.

**Figure 1:**
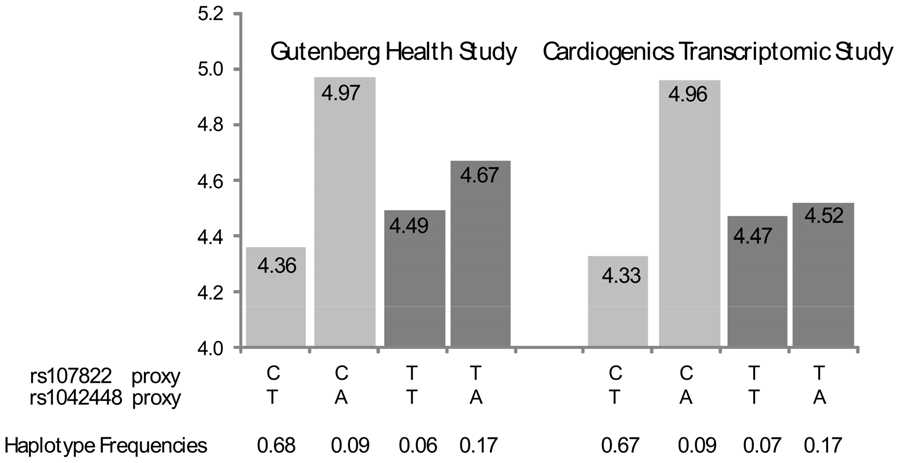
*HLA-DPB1* rs1042448 × *hsa-mir-219-1* rs107822 interaction on *HLA-DPB1* monocyte expression. In the Gutenberg Health Study, the rs1042248/rs107822 pair was tagged by rs3128923/rs213208. In the Cardiogenics Transcriptomic Study, the corresponding tagging pair was rs3117222/rs439205.

The first replicated interaction involved the *HLA-DPB1* rs1042448 and hsa-mir-219-1 rs107822 tagged by the rs3128923/rs213208 and rs3117222/rs439205 pairs in GHS and CTS, respectively. These two loci are distant from about 100 kb and the corresponding tag SNPs were in modest linkage disequilibrium (LD), r^2^ = 0.58 and r^2^ = 0.56, in GHS and CTS, respectively. In GHS, the haplotype analysis of the rs107822 and rs1042448 proxies revealed that the *HLA-DPB1* rs1042448-A proxy allele (i.e the allele at the proxy SNP that can be used to tag the rs1042448-A allele) was associated with a strong increase in *HLA-DBP1* expression (β = +0.61, p = 1.64×10^−105^) when carried on the same haplotype as the hsa-mir-219-1 rs107822-C proxy allele (Figure 1). Conversely, when associated with the hsa-mir-219-1 rs107822-T proxy allele, the increasing effect of the *HLA-DPB1* rs1042448-A proxy allele was significantly reduced (p = 1.88×10^−20^) and became β = +0.18 (p = 3.49×10^−8^) illustrating the interaction phenomenon identified through linear regression analysis. This interaction remained significant (p = 2.81×10^−12^) when the haplotype analysis was further adjusted on the best *cis* eSNP observed for *HLA-DBP1* expression, rs3128963 (p = 2.30×10^−151^) (see GHS_Express database [23]). The same pattern of associations was observed in CTS (Figure 1). The *HLA-DPB1* rs1042448-A proxy allele was associated with a strong significant increase in *HLA-DPB1* expression (β = +0.63, p = 5.24×10^−62^) when carried on the same haplotype as the hsa-mir-219-1 rs107822-C proxy allele. The corresponding increase when the rs1042248-A proxy allele was associated with the hsa-mir-219-1 rs107822-A proxy allele was significantly reduced (p = 2.68×10^−20^) and did no longer reach significance (β = +0.05; p = 0.23) (Figure 1).

**Figure 2:**
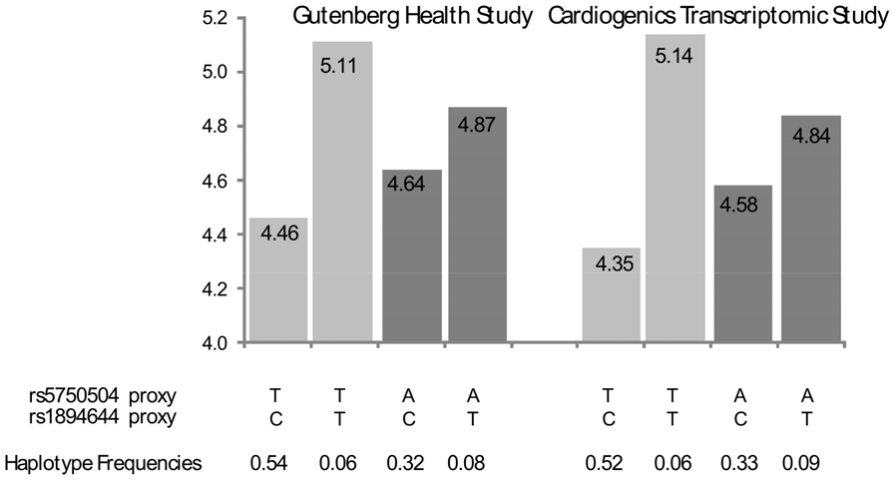
*H1F0* rs1894644 × *hsa-mir-659* rs5750504 interaction on *H1F0* monocyte expression. In the Gutenberg Health Study, the rs1894644/rs5750504 pair was tagged by rs763137/rs2899293. In the Cardiogenics Transcriptomic Study, the corresponding tagging pair was rs1894644/rs6000905.

The second replicated interaction involved the *H1F0* rs1894644 and hsa-mir-659 rs5750504 tagged by the rs763137/rs2899293 and rs1894644/rs6000905 pairs in GHS and CTS, respectively (Figure 2). These two loci are distant from about 40 kb and the corresponding tag SNPs were in low LD, r^2^ = 0.15 and r^2^ = 0.14, in GHS and CTS, respectively. In GHS and in CTS, the *H1F0* rs1894644-T proxy allele was associated with a strong increase in *H1F0* expression (β = +0.65, p = 1.71×10^−53^ and β = +0.79, p = 1.36×10^−40^, respectively) when it was on the same haplotype as the rs5750504-T proxy allele. Conversely, when the rs1894644-T proxy allele was on the same haplotype as the rs5750504-A proxy allele, the corresponding increase in *H1F0* expression was lower (β = +0.23, p = 9.74×10^−13^ and β = +0.26, p = 7.25×10^−8^, respectively). The test for homogeneity of the *H1F0* rs1894644 effect according to the rs5750504 proxy was significant p = 3.03×10^−12^ and p = 5.67×10^−10^ in GHS and CTS, respectively, validating the interaction detected through standard linear regression analysis (p = 2.98×10^−10^ and p = 1.37×10^−8^, respectively). Note that, in GHS, the rs763137 SNP involved in this interaction was the best *cis* eSNP for *H1F0* (p = 1.10×10^−62^).

As shown in Table S3, the two replicated interactions were consistent in CAD and healthy subjects composing CTS.

## Discussion

Coupling genome-wide association and expression studies have been an attractive strategy to disentangle the architecture of the genetics of gene expression and to assess whether gene expression dysregulation could mediate the effect of SNPs on disease risk identified through genome-wide association studies [23], [34]. To our knowledge, such studies [23], [34]–[37] mainly focused on assessing marginal associations of single SNPs with gene expression. Even if SNP × SNP interactions have often been advocated as a potential source of phenotype variability [38], [39], there has been few attempt to assess at the genome-wide scale whether such SNP × SNP interactions could influence gene expression variability. This is likely due to the statistical and computing burdens associated with such investigations characterized by a huge number of tested interactions and the very large sample size required to detect genome-wide significance. We postulated that focusing on plausible “biological” interactions could be one strategy to dig into the complex architecture of SNP × SNP interactions. This is why we undertook what we think is the first systematic and comprehensive search for interactions between SNPs located in the genomic sequence of miRNAs and SNPs located in the 3'UTR gene regions that could participate in monocyte gene expression. This search for interactions was preceded by a genome-wide investigation of miSNPs effect on monocyte expression to assess whether miSNPs could influence gene expression, in particular, through *trans* regulation.

These investigations were conducted in the Gutenberg Health Study where the extensive genome-wide study of marginal SNP associations with monocyte expressions had previously been reported and the results stored in a publicly available resource [23], and we replicated the significant findings in the Cardiogenics study.

Our survey of marginal miSNP effect has pointed out the hsa-mir-1279 miRNA mapping to chromosome 12q15 as a candidate regulator of 10 genes in monocytes. Indeed, we observed that the hsa-mir-1279 rs1463335 tagged by rs317657 or rs1463335 was robustly associated in *cis* with *LYZ* expression and in *trans* with *CNTN6*, *CTRC, COPZ2*, *KRT9*, *LRRFIP1*, *NOD1*, *PCDHA6*, *ST5* and *TRAF3IP2*. The bioinformatics prediction of the *LYZ* gene as a target for hsa-mir-1279 miRNA supports this hypothesis. The lack of strong correlation between the expression of these 10 genes, together with the *trans* association observed after adjusting for *LYZ* expression, could suggest that these nine genes could also be targets for the hsa-mir-1279, despite the absence of such prediction by current bioinformatics tools. However, the observation of positive associations with *LYZ* and *NOD1*, but of negative associations with the other genes, is puzzling as we could have expected, at first sight, a similar pattern of associations if all these genes were target for hsa-mir-1279. Functional experimental work is needed to characterize the role of hsa-mir-1279 in the regulation of these genes in-depth, in particular *TRAF3IP2* as this gene was identified in two independent GWAS as a susceptibility locus for psoriasis [40], [41]. Our results, if confirmed, could open therapeutics perspectives as it is possible to use artificial miRNA targets to modify gene expression [42], [43]. A *trans* association pattern was also recently observed at the locus 12q15 using an unsupervised gene networks analysis of the same datasets [24]. The rs11177644 located in the 3'UTR region of the *YEATS*4 gene was also found associated in *cis* to *LYZ* and *YEATS4* and in *trans* with a module of 36 genes including the *CNTN6*, *CTRC, COPZ2*, *KRT9*, *LRRFIP1*, *NOD1* and *ST5* discussed above. However, unlike what we observed here with hsa-mir-1279 rs1463335, the *trans* associations with rs11177644 had been found mediated by *cis* regulation mechanisms. Using a standard linear regression analysis (see above), we then tested whether these two SNPs could interact to contribute to the identified *trans* associations. We did not observe any strong evidence for such phenomenon as the lowest p-value for interaction was p = 8.53×10^−4^ for *PCDHA6* (data not shown). As the rs11177644 and rs1463335 were in moderate LD (r^2^ = 0.30 and D' = +0.70), we further conducted an haplotype analysis of the two SNPs (Table 6). This revealed that both SNPs acted additively on *LYZ* expression but, after adjusting for rs11177644, the association of rs1463335 with *YEATS4* was no longer significant (p = 0.748). This haplotype analysis also revealed strong *trans* haplotype associations, which were due to a single haplotype, (rs317657_C/rs11177644_A), which was, after adjusting for *LYZ* and *YEATS4* expression, strongly associated with increased levels of *NOD1* (p = 8.30×10^−13^), and decreased levels of the eight other genes, with p-values ranging from 2.21×10^−6^ to 2.52×10^−38^ (Table 6). These results suggest that the associations observed at the 12q15 locus are much more complex as initially hypothesized. It appeared that *YEATS4* and *LYZ* expressions could be under the influence of a common *cis* eSNP, but the latter would also be additionally influenced by a miSNP contributing to *trans* associations. As discussed in the following paragraph, further investigating including molecular experiments are required to dissect this complex pattern of association.

**Table 6:** Haplotype effects derived from the rs317657 and rs11177644 at the 12q15 locus in the Gutenberg Health Study (N = 1,467).

Polymorphisms		Haplotype Frequencies	Haplotype effects on Gene Expressions(1)					
rs317657	rs11177644		*YEATS4* (2)	*LYZ* (3)	*PCDHA6*	*LRRFIP1*	*ST5*	*KRT9*
C	A	0.399	reference	reference	reference	reference	reference	reference
C	G	0.061	−0.231[−0.271 – −0.191]	−0.209[−0.246 – −0.171]	+0.067[0.051 – 0.083]	+0.074[0.051 – 0.096]	+0.082[0.047 – 0.117]	+0.068[0.041 – 0.096]
T	A	0.155	+0.018[−0.008 – 0.044]	−0.073[−0.103 – −0.042]	+0.051[0.041 – 0.061]	+0.053[0.041 – 0.065]	+0.058[0.038 – 0.078]	+0.051[0.035 – 0.067]
T	G	0.385	−0.258[−0.277 – −0.240]	−0.281[−0.301 – −0.260]	+0.061[0.052 – 0.070]	+0.081[0.068 – 0.094]	+0.079[0.060 – 0.098]	+0.054[0.038 – 0.071]
Haplotypic association			R^2^ = 39.3%p = 1.72 10^−158^	R^2^ = 36.6%p = 7.08 10^−145^	R^2^ = 12.3%p = 2.52 10^−38^	R^2^ = 11.4%p = 1.00 10^−37^	R^2^ = 5.20%p = 3.02 10^−16^	R^2^ = 4.69%p = 2.55 10^−13^

Polymorphisms		Haplotype Frequencies	Haplotype effects on Gene Expressions(1)				
rs317657	rs11177644		*NOD1*	*CNTN6*	*CTRC*	*TRAF3IP2*	*COPZ2*
C	A	0.399	reference	reference	reference	reference	reference
C	G	0.061	−0.083[−0.120 – −0.045]	+0.017[0.004 – 0.029]	+0.031[0.011 – 0.051]	+0.022[0.007 – 0.037]	+0.029[0.002 – 0.056]
T	A	0.155	−0.033[−0.056 – −0.010]	+0.019[0.011 – 0.027]	+0.021[0.007 – 0.034]	+0.021[0.011 – 0.030]	+0.035[0.020 – 0.049]
T	G	0.385	−0.086[−0.108 – −0.064]	+0.023[0.016 – 0.031]	+0.039[0.028 – 0.051]	+0.024[0.015 – 0.033]	+0.028[0.014 – 0.042]
Haplotypic association			R^2^ = 4.08%p = 8.30 10^−13^	R^2^ = 3.31%p = 3.87 10^−10^	R^2^ = 3.08%p = 6.70 10^−10^	R^2^ = 2.87%p = 3.40 10^−8^	R^2^ = 2.27%p = 2.21 10^−6^

(1) Haplotype effects were estimated assuming haplotype additive effects after adjusting for age, gender, and *LYZ* and *YEATS4* expressions when appropriate.

(2) After adjusting for rs317657, the rs11177644-G allele was associated with decreased *YEATS4* expression (β = −0.262, p = 1.36 10^−138^). After adjusting for rs11177644, the rs317657-T allele was no longer associated with *YEATS4* expression (β = −0.003, p = 0.748).

(3) After adjusting for rs317657, the rs11177644-G allele was associated with decreased *LYZ* expression (β = −0.208, p = 9.56 10^−84^). After adjusting for rs11177644, the rs317657-T allele was associated with decreased *LYZ* expression (β = −0.072, p = 1.24 10^−10^).

Two interactions miSNPs × 3utrSNPs were robustly identified, the first involving *HLA-DPB1* rs1042448 and hsa-mir-219-1 rs107822, the second the *H1F0* rs1894644 and hsa-mir-659 rs5750504. In both cases, the identified 3'UTR rare alleles were found to strongly increase the expression of the associated genes, but these over-expressions were highly reduced in carriers of miSNPs rare alleles. The identified miSNPs are not located within the mature sequence of the associated miRNAs but in their pri-miRNA sequences. These rare alleles could either be associated with increased miRNA expression or could tag for yet-unknown miSNPs within mature sequences leading to the production of isomiRs. It could be speculated that the associated miRNAs or isomiRs would then target the identified 3'UTR regions made sensitive to miRNAs regulation by the identified 3'UTR variants, variants that could create novel motifs for miRNAs' binding and would lead to reduction of the *per se* effect of the 3'UTR variant. Molecular constructs are required to assess such hypothesis. We further checked whether the identified miSNPs could interact with other 3'UTR SNPs located in genes in the vicinity of the *HLA-DBP1* and *H1F0* loci. We did not observe any suggestive evidence (P<0.05) for such interaction suggesting that the identified miRNA regulation would be specific to *HLA-DBP1* and *H1F0*.

**Figure 3:**
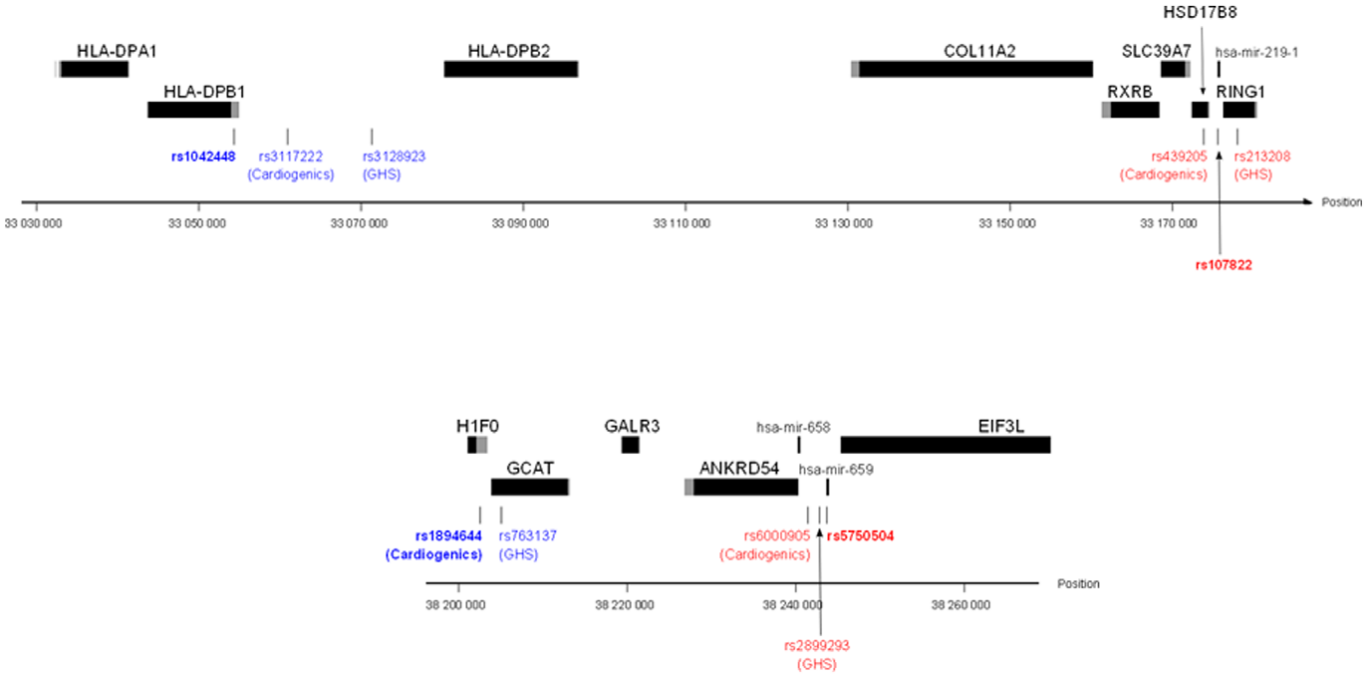
Location of genes, miSNP and 3'UTR SNPs at the two detected interacting loci. Gene are indicated as black rectangles with grey 3'UTR. Bold red and blue SNPs represent miSNPs and 3utrSNPs respectively. Corresponding proxies are non-bold coloured. Top: *HLA-DBP1* locus on chromosome 6; Bottom: *H1F0* locus on chromosome 22.

The identified interactions involved SNPs in modest LD but located within a genomic distance of less than 100 kb. Several examples have already been observed where a given miRNA participates to the regulation of a gene located in its very close vicinity [2], [3], [44], [45]. Nevertheless, one cannot exclude the possibility that the detected interactions are tagging for other complex haplotypic effects spanning a large distance and over several genes, five genes lying between *HLA-DPB1* and hsa-mir-219-1 and three between *H1F0* and hsa-mir-659 (Figure 3). Additional functional experiments would be required to biologically characterize the detected statistical interactions.

Little is known about *H1F0* in human diseases except that it codes for a histone family member protein. Interestingly, hsa-mir-659 has been shown to influence the risk of dementia [46] through a mechanism that could involve histone deacetylation [47], [48]. Although speculative, the joint contribution of *H1F0* and hsa-mir-659 on the risk of dementia could deserve further attention. Conversely the *HLA-DPB1* gene has been associated with several complex diseases such as pulmonary hypertension, hepatitis B infection and systemic sclerosis [49]–[51]. In addition, hsa-mir-219-1 was suggested to play a role in schizophrenia and in N-methyl-D-aspartate (NMDA) glutamate receptor signaling, two pathophysiological mechanisms linked to *HLA-DPB1* [52], [53] making our results of valuable information for scientists interested in these pathologies.

Several limitations of this work must be acknowledged. First, because our investigation was conducted on genotyped data of common SNPs, only 258 miRNAs were covered by our study, which represent less than one-quarter of the hypothesized total number of human miRNAs. Second, only one cell type was studied where not all genes are expressed. Therefore not all possible association could be explored. Third, expression were measured using the microarray technology that may be less efficient than emerging mRNA deep-sequencing methods for measuring, especially low abundant, mRNA levels [54], [55]. Because a given miRNA can bind several genes and a given 3'UTR can be a target for several miRNAs, compensation phenomena are proposed to explain the relative low impact of miRNA regulation on mRNA expression generally observed [56]. Therefore, genetic effects associated with miRNA and 3'UTR SNPs are hypothesized to be a modest size and very large sample size would be required to detect them. Despite having robustly identified two interactions, we cannot then exclude that other interactions with lower magnitude could have been missed due to power considerations, even if the two genome-wide expression datasets used in this work are among the largest collected so far in human epidemiological studies. Third, by discarding from our investigations probes harboring a SNP in their genomic sequence to avoid any bias in the results of the association analyses, some miRNA-sensitive regulatory mechanisms associated to genes tagged by probes matching their 3'UTR region may have been missed. Last, our investigation was conducted in monocytes and results observed may not be portable to other cells or tissues.

Nevertheless, our study illustrates that the proposed strategy searching for interaction between miSNPs and 3'UTR SNPs in genome-wide expression studies could be an alternative to bioinformatics prediction tools to identify miRNA targeted genes.

## Materials and Methods

### Ethics Statement

This work was based on two genome-wide expression studies, the Gutenberg Health Study (GHS) for the discovery phase and the Cardiogenics Transcriptomic Study (CTS) for the replication stage. Both studies were approved by the Institutional Ethical Committee of each participating center and by the local and federal data safety commissioners (Ethik-Kommission der Landesärztekammer Rheinland-Pfalz) for GHS. These two studies have already been extensively described in [21]–[23] for GHS and in [26], [57] for CTS.

### Gutenberg Health Study

This analysis was conducted in a population-based sample of 750 men and 717 women aged 35–74 years, of European descent. Monocytic RNA was isolated from peripheral blood monocytes by negative selection using RosetteSep Monocyte Enrichment Cocktail (StemCell Technologies, Vancouver, Canada), Trizol extraction and purification by silica-based columns. Expression profiles were assessed using the *Illumina* HT-12 v3 BeadChip (Illumina, CA, USA) with ∼48,000 probes covering 37,804 genes, and generated data were pre-processed using Beadstudio. Values from probes with ≤1 bead were re-imputed using SVD impute from the pcaMethods R package [58]. Data were normalized using quantile normalization and VST transformation as implemented in the lumi R package. To avoid spurious associations due to hybridation difference, probes that contained SNPs or were not annotated to be of “*perfect*” quality according to ReMOAT [28] (Reannotation and Mapping of Oligonucleotide Arrays Technologies, http://remoat.sysbiol.cam.ac.uk) were discarded. Individuals were typed for genome-wide genotype data using the Affymetrix Genome-Wide Human SNP Array 6.0 (Affymetrix, CA, USA). SNP analysis was restricted to autosomal SNPs with minor allele frequency >0.01, call rate >0.98 and Hardy-Weinberg equilibrium testing p-value >10^−4^.

### Cardiogenics Study

The present study included monocyte expression data from 758 individuals from European descent, 363 patients with coronary artery disease and 395 unrelated healthy individuals.

Monocyte RNAs were isolated from whole blood using CD14 micro beads (Miltenyi) and expression profile was processed in a single center using the *Illumina* HumanRef-8 v3 beadchip array (*Illumina* Inc., San Diego, CA) containing 24,516 probes corresponding to 18,311 distinct genes. After hybridization, array images were scanned using the *Illumina* BeadArray Reader and probe intensities were extracted using the Gene expression module (version 3.3.8) of the *Illumina BeadStudio* software (version 3.1.30). Raw intensities were processed in R statistical environment using the Lumi and beadarray packages. All array outliers were excluded and only arrays with high concordance in terms of gene expression measures (pairwise Spearman correlation coefficients within each cell type >0.85) were included in the analyses.

Genomic DNA was extracted from peripheral blood leucocytes by standard procedures (Qiagen). Genome-wide genotyping was carried out using one of two *Illumina* arrays; the Sentrix Human Custom 1.2 M array and the Human 610 Quad Custom array. Data from the two arrays was combined as described in [59]. SNP analysis was restricted to autosomal SNPs with minor allele frequency >0.01, call rate >0.95 and Hardy-Weinberg equilibrium testing p-value >10^−5^.

### Statistical analysis

The association of miSNP proxies with probe expression was tested by use of a standard linear regression model under the assumption of additive allele effects (i.e. proxy genotype coded as 0/1/2 according the number of rare alleles). Pair-wise SNPs interactions on probe expression were tested using a standard linear regression model in which both SNP (miSNP and 3utrSNP) genotypes were coded as 0,1,2 together with the corresponding product term for interaction. All analyses were adjusted for age and gender, and additionally for disease status in CTS.

In the Gutenberg Health Study, a weighted-Bonferroni procedure was applied to identify genome-wide significant interactions. Each 3utrSNP was first assessed using the Levene statistic [29] testing the equality of associated-probe expression variance across genotypes. The resulting log(p-value) was then used to weight the interaction p-value obtained from the linear regression analysis. This strategy is expected to be more powerful than a standard Bonferroni correction procedure [60], [61] as it gives more weight to interaction involving probes showing higher differences in inter-genotype variance.

For each 3utrSNP *u* (*u* = 1 to N_utr_) associated with a Levene test p-value q*_u_*, we define a standardized weight *w_u_* as


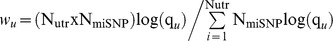
 such as 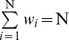 where N_utr_, N_miSNP_, N are the total number of studied 3utrSNPs, miSNPs and interactions, respectively. Each interaction p-value P*_i_* is then weighted by the w*_u_* corresponding to the 3utrSNP that is involved in the interaction, leading to a weighted p-value P_i_*. Each Pi* that is then below 0.05/N is then declared genome-wide significant at the 0.05 type I error.

In Cardiogenics, the standard Bonferroni threshold was used to declare significance.

Identified interactions between pairs of SNPs were illustrated through haplotype analyses conducted by the THESIAS software implementing a Stochastic-EM algorithm for haplotype-based association analysis [62]. All other statistical analyses were performed in R v. 2.12.0.

## Supporting Information

Table S1 **Genome-wide significant (p<7.7 10^−9^) associations of miSNPs on monocyte gene expression in the Gutenberg Health Study.** ^(1)^ SNPs showing the strongest association (with P<5 10^−5^) with gene expression within 1Mb of the associated probe. ^(2)^ Regression coefficient associated with the rare miSNP allele under an additive effect model, adjusted for age and gender. ^(3)^ P-value of the association between miSNP and gene expression. ^(4)^ P-value of the association between miSNP and gene expression adjusted for the best *cis* eSNP. ^(5)^ Pairwise r2 between miSNP and best *cis* eSNPs in GHS. ^(6)^ The best *cis* eSNP and the associated-miSNP coincide.
(XLSX)

Table S2 ***Cis*** **and** ***trans*** **-associations observed with the hsa-mir-1279 rs1463335^(1)^ separately in CAD patients and healthy subjects of the Cardiogenics Transcriptomic Study.** ^(1)^ The rs1463335 was tagged by the rs998022 in CTS. The rs146335 is located on chromosome 12, at position 69,667,075. As a consequence, the association observed with LYZ and YEATS4 are considered as *cis*-associations, the remaining eight as trans-associations. ^(2)^ Regression coefficient associated with the rare miSNP allele under an additive effect model, adjusted for age and gender. ^(3)^ P-value of the association between miSNP and gene expression.
(DOCX)

Table S3 **Patterns of detected miSNPs × 3utrSNPs interaction separately in CAD and healthy subjects of the Cardiogenics Transcriptomic Study.** ^(1)^ Regression coefficient of the interaction term when both miSNP and 3utr proxy SNPs coded 0/1/2 according to the number of carried rare alleles are introduced in a linear regression model together with their interaction term. ^(2)^ P-value of the interaction test derived from the standard linear regression analysis in CTS. Bold p-values correspond to the detected interactions that were significant after Bonferroni correction in the whole CTS.
(DOCX)
