# Correction: Comprehensive Exploration of the Effects of miRNA SNPs on Monocyte Gene Expression

**DOI:** 10.1371/annotation/bd5c0312-c902-4065-be2b-385fcf70c125

**Published:** 2012-10-05

**Authors:** Nicolas Greliche, Tanja Zeller, Philipp S. Wild, Maxime Rotival, Arne Schillert, Andreas Ziegler, Cardiogenics Consortium, Tony Attwood, Belz Stephanie, Peter Braund, Jessy Brocheton, Jason Cooper, Abi Crisp-Hihn, Patrick (formerly Linsel-Nitschke) Diemert, Nicola Foad, Tiphaine Godefroy, Jay Gracey, Emma Gray, Rhian Gwilliams, Susanne Heimerl, Jennifer Jolley, Unni Krishnan, Heather Lloyd-Jones, Ulrika Liljedahl, Ingrid Lugauer, Per Lundmark, Seraya Maouche, Jasbir S Moore, Montalescot Gilles, David Muir, Elizabeth Murray, Chris P Nelson, Jessica Neudert, David Niblett, Karen O’Leary, Helen Pollard, Carole Proust, Angela Rankin, Augusto Rendon, Catherine M Rice, Hendrik Sager, Jennifer Sambrook, Schmitz Gerd, Michael Scholz, Laura Schroeder, Jonathan Stephens, Ann-Christine Syvannen, Stefanie (formerlyGulde) Tennstedt, Chris Wallace, Panos Deloukas, Jeanette Erdmann, Christian Hengstenberg, Willem H. Ouwehand, Nilesh J. Samani, Heribert Schunkert, Thomas Munzel, Karl J. Lackner, François Cambien, Alison H. Goodall, Laurence Tiret, Stefan Blankenberg, David-Alexandre Trégouët

**Affiliations:** Department of Haematology, University of Cambridge, Long Road, Cambridge, CB2 2PT, UK and National Health Service Blood and Transplant, Cambridge Centre, Long Road, Cambridge, CB2 2PT, UK; Medizinische Klinik 2, Universität zu Lübeck, Lübeck Germany; Department of Cardiovascular Sciences, University of Leicester, Glenfield Hospital, Groby Road, Leicester, LE3 9QP, UK; INSERM UMRS 937, Pierre and Marie Curie University (UPMC, Paris 6) and Medical School, 91 Bd de l’Hôpital 75013, Paris, France; Juvenile Diabetes Research Foundation/Wellcome Trust Diabetes and Inflammation Laboratory, Department of Medical Genetics, Cambridge Institute for Medical Research, University of Cambridge, Wellcome Trust/MRC Building, Cambridge, CB2 0XY, UK; Department of Haematology, University of Cambridge, Long Road, Cambridge, CB2 2PT, UK and National Health Service Blood and Transplant, Cambridge Centre, Long Road, Cambridge, CB2 2PT, UK; Medizinische Klinik 2, Universität zu Lübeck, Lübeck Germany; Department of Haematology, University of Cambridge, Long Road, Cambridge, CB2 2PT, UK and National Health Service Blood and Transplant, Cambridge Centre, Long Road, Cambridge, CB2 2PT, UK; INSERM UMRS 937, Pierre and Marie Curie University (UPMC, Paris 6) and Medical School, 91 Bd de l’Hôpital 75013, Paris, France; Department of Cardiovascular Sciences, University of Leicester, Glenfield Hospital, Groby Road, Leicester, LE3 9QP, UK; The Wellcome Trust Sanger Institute, Wellcome Trust Genome Campus, Hinxton, Cambridge CB10 1SA, UK; The Wellcome Trust Sanger Institute, Wellcome Trust Genome Campus, Hinxton, Cambridge CB10 1SA, UK; Klinik und Poliklinik für Innere Medizin II, Universität Regensburg, Germany; Department of Haematology, University of Cambridge, Long Road, Cambridge, CB2 2PT, UK and National Health Service Blood and Transplant, Cambridge Centre, Long Road, Cambridge, CB2 2PT, UK; Department of Cardiovascular Sciences, University of Leicester, Glenfield Hospital, Groby Road, Leicester, LE3 9QP, UK; Department of Haematology, University of Cambridge, Long Road, Cambridge, CB2 2PT, UK and National Health Service Blood and Transplant, Cambridge Centre, Long Road, Cambridge, CB2 2PT, UK; Molecular Medicine, Department of Medical Sciences, Uppsala University, Uppsala, Sweden; Klinik und Poliklinik für Innere Medizin II, Universität Regensburg, Germany; Molecular Medicine, Department of Medical Sciences, Uppsala University, Uppsala, Sweden; Medizinische Klinik 2, Universität zu Lübeck, Lübeck Germany; INSERM UMRS 937, Pierre and Marie Curie University (UPMC, Paris 6) and Medical School, 91 Bd de l’Hôpital 75013, Paris, France; Department of Cardiovascular Sciences, University of Leicester, Glenfield Hospital, Groby Road, Leicester, LE3 9QP, UK; INSERM UMRS 937, Pierre and Marie Curie University (UPMC, Paris 6) and Medical School, 91 Bd de l’Hôpital 75013, Paris, France; Department of Haematology, University of Cambridge, Long Road, Cambridge, CB2 2PT, UK and National Health Service Blood and Transplant, Cambridge Centre, Long Road, Cambridge, CB2 2PT, UK; Department of Haematology, University of Cambridge, Long Road, Cambridge, CB2 2PT, UK and National Health Service Blood and Transplant, Cambridge Centre, Long Road, Cambridge, CB2 2PT, UK; Department of Cardiovascular Sciences, University of Leicester, Glenfield Hospital, Groby Road, Leicester, LE3 9QP, UK; Trium, Analysis Online GmbH, Hohenlindenerstr. 1, 81677, München, Germany; The Wellcome Trust Sanger Institute, Wellcome Trust Genome Campus, Hinxton, Cambridge CB10 1SA, UK; Department of Haematology, University of Cambridge, Long Road, Cambridge, CB2 2PT, UK and National Health Service Blood and Transplant, Cambridge Centre, Long Road, Cambridge, CB2 2PT, UK; Department of Cardiovascular Sciences, University of Leicester, Glenfield Hospital, Groby Road, Leicester, LE3 9QP, UK; INSERM UMRS 937, Pierre and Marie Curie University (UPMC, Paris 6) and Medical School, 91 Bd de l’Hôpital 75013, Paris, France; Department of Haematology, University of Cambridge, Long Road, Cambridge, CB2 2PT, UK and National Health Service Blood and Transplant, Cambridge Centre, Long Road, Cambridge, CB2 2PT, UK; European Bioinformatics Institute, Wellcome Trust Genome Campus, Hinxton, Cambridge, CB10 1SD, UK; The Wellcome Trust Sanger Institute, Wellcome Trust Genome Campus, Hinxton, Cambridge CB10 1SA, UK; Medizinische Klinik 2, Universität zu Lübeck, Lübeck Germany; Department of Haematology, University of Cambridge, Long Road, Cambridge, CB2 2PT, UK and National Health Service Blood and Transplant, Cambridge Centre, Long Road, Cambridge, CB2 2PT, UK; Institut für KlinischeChemie und Laboratoriums medizin, Universität, Regensburg, D-93053 Regensburg, Germany; Trium, Analysis Online GmbH, Hohenlindenerstr. 1, 81677, München, Germany; Medizinische Klinik 2, Universität zu Lübeck, Lübeck Germany; Department of Haematology, University of Cambridge, Long Road, Cambridge, CB2 2PT, UK and National Health Service Blood and Transplant, Cambridge Centre, Long Road, Cambridge, CB2 2PT, UK; Molecular Medicine, Department of Medical Sciences, Uppsala University, Uppsala, Sweden; Medizinische Klinik 2, Universität zu Lübeck, Lübeck Germany; Juvenile Diabetes Research Foundation/Wellcome Trust Diabetes and Inflammation Laboratory, Department of Medical Genetics, Cambridge Institute for Medical Research, University of Cambridge, Wellcome Trust/MRC Building, Cambridge, CB2 0XY, UK

There was an error in the author byline.

The correct byline is: Nicolas Greliche^1,2^, Tanja Zeller^3^, Philipp S. Wild^4^, Maxime Rotival^1¤^, Arne Schillert^5^, Andreas Ziegler^5^, the Cardiogenics Consortium^¶^, Panos Deloukas^6^, Jeanette Erdmann^7^, Christian Hengstenberg^8^, Willem H. Ouwehand^6,9^, Nilesh J. Samani^10,11^, Heribert Schunkert^7^, Thomas Munzel^4^, Karl J. Lackner^12^, François Cambien^1^, Alison H. Goodall^10,11^, Laurence Tiret^1^, Stefan Blankenberg^3^, David-Alexandre Trégouët^1,13*^

