# Supplementary material for: Comprehensive Exploration of the Effects of miRNA SNPs on Monocyte Gene Expression
Source: PLoS One. 2012 Sep 21;7(9):e45863. doi: 10.1371/journal.pone.0045863 (PMC3448685; doi:10.1371/journal.pone.0045863)
Supplement: Table S2 — Cis and trans -associations observed with the hsa-mir-1279 rs1463335(1) separately in CAD patients and healthy subjects of the Cardiogenics Transcriptomic Study. (1) The rs1463335 was tagged by the rs998022 in CTS. The rs146335 is located on chromosome 12, at position 69,667,075. As a consequence, the association observed with LYZ and YEATS4 are considered as cis-associations, the remaining eight as trans-associations. (2) Regression coefficient associated with the rare miSNP allele under an additive effect model, adjusted for age and gender. (3) P-value of the association between miSNP and gene expression. (DOCX) [file pone.0045863.s002.docx]

**Table S2 *Cis* and *trans*-associations observed with the hsa-mir-1279 rs1463335^(1)^ separately in CAD patients and healthy subjects of the Cardiogenics Transcriptomic Study.**

| Associated Gene Expression | | | | | CAD | | | Healthy | | |
| --- | --- | --- | --- | --- | --- | --- | --- | --- | --- | --- |
| Probe | Gene | CHR | Start | End | β^(2)^ | SE | P^(3)^ | β^(2)^ | SE | P^(3)^ |
| ILMN_1748730 | CTRC | 1 | 15764937 | 15773152 | -0.05 | 0.01 | 1.5 10^-6^ | -0.07 | 0.01 | 9.0 10^-11^ |
| ILMN_2252021 | LRRFIP1 | 2 | 238536223 | 238690289 | -0.11 | 0.01 | 9.6 10^-14^ | -0.14 | 0.01 | 1.0 10^-20^ |
| ILMN_1699317 | CNTN6 | 3 | 1134628 | 1445277 | -0.03 | 0.01 | 8.2 10^-6^ | -0.04 | 0.01 | 3.3 10^-7^ |
| ILMN_1740494 | PCDHA6 | 5 | 140207649 | 140391928 | -0.08 | 0.01 | 4.8 10^-11^ | -0.12 | 0.01 | 3.1 10^-23^ |
| ILMN_1663381 | TRAF3IP2 | 6 | 111880142 | 111927320 | -0.06 | 0.01 | 4.9 10^-8^ | -0.07 | 0.01 | 5.7 10^-11^ |
| ILMN_2114422 | NOD1 | 7 | 30464142 | 30518392 | 0.11 | 0.02 | 5.1 10^-10^ | 0.12 | 0.02 | 2.2 10^-11^ |
| ILMN_1731063 | ST5 | 11 | 8714898 | 8932497 | -0.20 | 0.03 | 2.2 10^-12^ | -0.25 | 0.03 | 6.5 10^-21^ |
| ILMN_1815205 | LYZ^(1)^ | 12 | 69742133 | 69748012 | NA | NA | NA | NA | NA | NA |
| ILMN_1801387 | YEATS4^(1)^ | 12 | 69753531 | 69784575 | 0.20 | 0.03 | 2.4 10^-10^ | 0.18 | 0.02 | 9.7 10^-13^ |
| ILMN_1792568 | KRT9 | 17 | 39722092 | 39728309 | -0.12 | 0.02 | 2.3 10^-7^ | -0.10 | 0.02 | 3.6 10^-6^ |
| ILMN_1667361 | COPZ2 | 17 | 46103532 | 46115151 | -0.09 | 0.02 | 8.1 10^-8^ | -0.12 | 0.02 | 5.3 10^-13^ |
