# Supplementary material for: Comprehensive Exploration of the Effects of miRNA SNPs on Monocyte Gene Expression
Source: PLoS One. 2012 Sep 21;7(9):e45863. doi: 10.1371/journal.pone.0045863 (PMC3448685; doi:10.1371/journal.pone.0045863)
Supplement: Table S3 — Patterns of detected miSNPs × 3utrSNPs interaction separately in CAD and healthy subjects of the Cardiogenics Transcriptomic Study. (1) Regression coefficient of the interaction term when both miSNP and 3utr proxy SNPs coded 0/1/2 according to the number of carried rare alleles are introduced in a linear regression model together with their interaction term. (2) P-value of the interaction test derived from the standard linear regression analysis in CTS. Bold p-values correspond to the detected interactions that were significant after Bonferroni correction in the whole CTS. (DOCX) [file pone.0045863.s003.docx]

**Table S3 Patterns of detected miSNPs x 3utrSNPs interaction separately in CAD and healthy subjects of the Cardiogenics Transcriptomic Study**

| miSNP x 3utrSNP | rs17349873 rs2278768 | rs107822 rs1042448 | rs257095 rs2278768 | rs5750504 rs1894644 | rs6963819 rs10473 | rs262404 rs1044561 | rs2284385 rs6060539 | rs257095 rs1044561 |
| --- | --- | --- | --- | --- | --- | --- | --- | --- |
| miRNA (CHR) | hsa-mir-3119-1 (1) | hsa-mir-219-1 (6) | hsa-mir-4636 (5) | hsa-mir-659 (22) | hsa-mir-490 (7) | hsa-mir-3973 (11) | hsa-mir-4755 (20) | hsa-mir-4636 (5) |
| Gene (CHR) | ASB1 (2) | HLA-DPB1 (6) | ASB1 (2) | H1F0 (22) | MXRA7 (7) | ASB1 (2) | RBM12 (20) | ASB1 (2) |
| Probe | ILMN_1683096 | ILMN_1749070 | ILMN_1683096 | ILMN_1757467 | ILMN_1743836 | ILMN_1683096 | ILMN_1670841 | ILMN_1683096 |
| CAD | | | | | | | | |
| Proxies | rs1330387 rs2278768 | rs213208 rs3128923 | rs6555591 rs2278768 | rs2899293 rs763137 | rs2350780 rs7221855 | rs16928224 rs2334004 | rs2284390 rs2425125 | rs6555591 rs2334004 |
| β^(1)^ | 0.04 | -0.25 | 0.02 | -0.26 | 0.00 | -0.14 | 0.09 | 0.02 |
| P-value ^(2)^ | 0.83 | **6.6 10^-6^** | 0.89 | 1.4 10^-4^ | 0.98 | 0.37 | 0.21 | 0.89 |
| Healthy | | | | | | | | |
| Proxies | rs6703198 rs10084192 | rs439205 rs3117222 | rs257095 rs10084192 | rs6000905 rs1894644 | rs2350780 rs9910052 | rs262407 rs10084192 | rs2038123 rs6121015 | rs257095 rs10084192 |
| β^(1)^ | 0.30 | -0.29 | 0.07 | -0.25 | 0.02 | 0.11 | 0.10 | 0.07 |
| P-value ^(2)^ | 0.23 | **8.9 10^-9^** | 0.44 | **1.0 10^-4^** | 0.53 | 0.50 | 0.24 | 0.44 |
